# Mechanistic movement models identify continuously updated autumn migration cues in Arctic caribou

**DOI:** 10.1186/s40462-021-00288-0

**Published:** 2021-11-01

**Authors:** Matthew D. Cameron, Joseph M. Eisaguirre, Greg A. Breed, Kyle Joly, Knut Kielland

**Affiliations:** 1 Department of Biology and Wildlife, University of Alaska Fairbanks, 2090 Koyukuk Drive, Fairbanks, AK 99775 USA; 2 Gates of the Arctic National Park and Preserve, Arctic Inventory and Monitoring Network, National Park Service, 4175 Geist Road, Fairbanks, AK 99709 USA; 3 Institute of Arctic Biology, University of Alaska Fairbanks, 2140 Koyukuk Drive, Fairbanks, AK 99775 USA; 4 Present Address: U.S. Fish and Wildlife Service, Marine Mammals Management, 1011 E. Tudor Rd., Anchorage, AK 99503 USA

**Keywords:** Arctic, Bayesian, Caribou, Correlated random walk, Migration cues, Migratory pacing, Movement ecology, *Rangifer tarandus*, Recursive Bayesian computation, Stopover

## Abstract

**Background:**

Migrations in temperate systems typically have two migratory phases, spring and autumn, and many migratory ungulates track the pulse of spring vegetation growth during a synchronized spring migration. In contrast, autumn migrations are generally less synchronous and the cues driving them remain understudied. Our goal was to identify the cues that migrants use in deciding when to initiate migration and how this is updated while *en route*.

**Methods:**

We analyzed autumn migrations of Arctic barren-ground caribou (*Rangifer tarandus*) as a series of persistent and directional movements and assessed the influence of a suite of environmental factors. We fitted a dynamic-parameter movement model at the individual-level and estimated annual population-level parameters for weather covariates on 389 individual-seasons across 9 years.

**Results:**

Our results revealed strong, consistent effects of decreasing temperature and increasing snow depth on migratory movements, indicating that caribou continuously update their migratory decision based on dynamic environmental conditions. This suggests that individuals pace migration along gradients of these environmental variables. Whereas temperature and snow appeared to be the most consistent cues for migration, we also found interannual variability in the effect of wind, NDVI, and barometric pressure. The dispersed distribution of individuals in autumn resulted in diverse environmental conditions experienced by individual caribou and thus pronounced variability in migratory patterns.

**Conclusions:**

By analyzing autumn migration as a continuous process across the entire migration period, we found that caribou migration was largely related to temperature and snow conditions experienced throughout the journey. This mechanism of pacing autumn migration based on indicators of the approaching winter is analogous to the more widely researched mechanism of spring migration, when many migrants pace migration with a resource wave. Such a similarity in mechanisms highlights the different environmental stimuli to which migrants have adapted their movements throughout their annual cycle. These insights have implications for how long-distance migratory patterns may change as the Arctic climate continues to warm.

**Supplementary Information:**

The online version contains supplementary material available at 10.1186/s40462-021-00288-0.

## Background

Movement is a fundamental adaptation by animals and migration is a prime example thereof to improve fitness in environments characterized by seasonally predictable spatiotemporal fluctuations in conditions [1–3]. The spatial scale of migration can vary drastically among or even within taxa. Regardless of distance, however, a complete migration trajectory is composed of a series of persistent and directional movements that emerge from a complex suite of physiological and behavioral adaptations [4, 5]. An integral component for understanding how migratory patterns arise is to identify the cues that migrants use in deciding when to initiate migration and how to move while *en route*.

For many species, spring migrations are tightly linked to seasonal resource pulses [6–10]. Under the Green Wave Hypothesis, migrants track fronts of emergent, high-quality vegetation to increase nutrient intake as spring progresses along the migratory route [11, 12]. Implicit to this paradigm of spring migration is that herbivores track these emergent vegetative fronts based on the perception of proximate resource quality, permitting migrants to move with resource gradients along the migration route. This is applicable to both temperate migrants in spring and tropical migrants at the beginning of the wet season [13–15]. It does not, however, explain fall migrations in temperate migrants, some Arctic migrants in spring, nor the transition to the dry season for tropical migrants [e.g., 16]. Learning can influence migration and some migrants use their memory more than perception of proximal cues to navigate to distant destinations [17–20]. Regardless of the relative influence of reactive (perception-based) and proactive (learned) mechanisms in driving migration behavior, birthing generally coincides with peak resource quality [5, 21], and this likely constrains variability and enhances synchronization of spring migration timing and pace [16, 22, 23].

In contrast to spring migration, autumn migration has been less studied and lacks a common, driving life history event (i.e. birthing) across taxa. Unlike the distinct pulse of vegetation green-up of spring, senescence of vegetation in autumn is prolonged and marked by a gradual decline of forage quality [24]. Perhaps owing to the greater observed variability in autumn phenology patterns during this time, factors influencing autumn migration have received but a fraction of the attention in research on spring migration [25] and autumn migration research still lacks a consistent theoretical framework across taxa.

For temperate ungulate species, vegetative productivity, snow, and temperature influence autumn migration to varying degrees. For example, autumn migrations in roe deer (*Capreolus capreolus*) and red deer (*Cervus elaphus*) populations across Europe were influenced by decreased vegetation productivity but not snow events [26]. For red deer in Norway, most individuals left the summer range before the first snowfall, but snow appeared to trigger autumn migration for those that remained [27]. Moreover, migration initiation was associated with decreasing temperatures for females [27] but not with vegetation senescence for either sex [22]. Snow interacts with decreasing temperature in white-tailed deer (*Odocoileus virginianus*) autumn migrations, such that the first snow to occur in colder temperatures greatly increased the likelihood of migration [28]. Snow and temperature have similar effects on the timing of autumn migration for mule deer [23, 29, 30]. These studies were all conducted for short-distance migrants and it is unclear if these relationships hold for long-distance migrants.

Populations of barren-ground caribou exhibit the longest terrestrial, non-volant migrations on the planet, for which round-trip distances between seasonal ranges can reach 1,350 km [31]. Despite a long history of interest in the drivers of autumn migration in caribou, contemporary research on the topic is surprisingly sparse. In 1913, the early naturalist Arthur Dugmore [32] speculated that the winter’s first heavy snowfall and cold temperatures initiated autumn migration for Newfoundland caribou based on local observations. Early fieldwork in Canada drew a connection between the first snowfall and autumn migration [33] and later work in Newfoundland suggested that the first snowfall of 5–10 cm initiated autumn migrations [34]. Anecdotally, autumn migration for the Porcupine Herd in Alaska and far western Canada was once observed to begin following an early season (August) snowstorm [35]. Many of these early field observations also note that autumn migrations slow down or pause altogether if the weather turns mild after such snow events, but resumed when temperatures decreased or snow began to accumulate again [32, 34, 35]. More recently, temporal variation in autumn migration for the George River and Leaf River Herds in northern Canada was linked to conditions *en route*, with earlier arrival at the winter range associated with deeper snow at the destination [36]. A promising avenue of migration research is to precisely determine how long-distance terrestrial migrants, such as caribou, respond to experienced environmental conditions throughout migration, given inherent annual environmental variability and the dispersed nature of caribou groups in autumn [33].

Previous studies have typically treated autumn migration, and the initiation of it, as a single discrete event, and applied analyses designed to relate environmental covariates to the timing of the start and end of migrations [e.g., 23, 27, 37, 38]. This approach explicitly assumes that once initiated, migration continues to its completion. Yet, many long-distance avian migrants [21], as well as migratory ungulates such as mule deer [38], red deer [22], and elk (*Cervus canadensis*) [39], use stopovers (pauses along the migration route) to replenish reserves, sometimes for many weeks. A promising new concept that incorporates variability in movement along a complete migration trajectory is “migratory pacing,” in which an individual continuously adjusts its behavior based on environmental conditions experienced *en route* [40]. Migratory pacing incorporates stopovers as an example of a distinct change in migration behavior in response to resources, while also incorporating more subtle changes in movement such as different movement rates. Green wave surfing in spring is an example of this behavior in ungulates, in which migrants pace migration to match the wave of spring resource quality [6, 10]. In contrast, how this concept applies to autumn migration in ungulates remains largely unexplored despite a long history of field observations and anecdotes suggesting a similar pacing-type pattern in many taxa. Recent developments in statistical movement models permit characterizing behavioral indices from GPS location data [41] and enables relating these behavioral states to experienced environmental conditions [40, 42].

We propose that a complete seasonal migration consists of a series of persistent, directional movements (hereafter simply “migratory movements”), that may or may not be interspersed with bouts of non-persistent movement (akin to stopovers) occurring at the individual level [1, 5]. We test for effects of continuously varying environmental characteristics on autumn migratory movements evaluated as dynamic parameters of a correlated random walk movement model. We examine these metrics in the Western Arctic Herd, a population of migratory, barren-ground caribou in northwest Alaska. We combine recently developed methods to test for effects at the individual level and scale these insights up for population-level inference [40, 43]. We hypothesize that (1) autumn migratory movements for caribou are a function of contemporaneous, experienced environmental conditions, (2) migration is paced based on a continuous decision-making process, such that if conditions change, movements are accelerated, adjusted, or paused, and (3) these responses are highly consistent throughout the population and across the study period despite the widely disaggregated nature of caribou in autumn. As we show, environmental conditions that are strongly affected by climate change alter migratory behavior, and we discuss our findings in relation to the potential for a continued change to alter long-distance terrestrial migrations in the Arctic.

## Material and methods

### Study population

We analyzed data from 175 individual collared caribou from the Western Arctic Herd in northwest Alaska, which annually range from approximately 65°–71° N and 166°–150° W. In autumn, the herd generally migrates from the arctic tundra of Alaska’s North Slope, through the rugged Brooks Range with peaks over 2000 m, to lichen-rich uplands and boreal forests south of the mountain range where they spend the winter. Wintering areas vary by year and the herd is typically broadly dispersed at this time [44, 45]. Autumn migration timing varies by year as well, with a trend toward later migration in recent years and proportions of the population not fully migrating south [44, 46]. From 2009 to 2018, GPS collars (Telonics, Mesa, AZ) were deployed on adult females using procedures approved by the State of Alaska Institutional Animal Care and Use Committee (permits 2012-031R and 0040-2017-40). Deployments occurred during autumn migration as caribou crossed the Kobuk River in Kobuk Valley National Park. Methods for collar deployment are described elsewhere [47, 48]. Most collars were set to record locations every 8 h, but some more recently deployed collars recorded locations every 2 or 4 h. For our analysis, the data were subsampled to 8-h location intervals for consistency across all individuals and years. During the study period, the herd size decreased from 355,000 animals in 2009 [46] to 244,000 in 2019 [49].

We analyzed GPS data between August 15 and December 31 for 2010–2018, resulting in nine autumn migration periods. We used 389 individual-season datasets across these 9 years, ranging from 28 in 2010 to 66 in 2016 (Additional File 1: Table S1). Movement rates associated with insect avoidance in mid-summer are the greatest of the year [46, 50]; consequently, we used August 15th as the beginning date for the analysis period based on preliminary investigations of the data which suggested that insect harassment season could extend to mid-August. Winter is characterized by the slowest and most localized movements of the year, and migration is complete by the end of the year [46, 50], so we ended the analysis period at the end of December. We only used data for which the collar was active for the entire period, and thus excluded individual-seasons where the individual died, was collared during the migratory period, or for which the individual had less than half of the expected GPS locations due to missing collar data (often due to poor satellite network connectivity).

### Movement model

To characterize the behavior of each caribou along its GPS movement track and understand how it was related to contemporaneous environmental factors the individual experienced, we fit a continuous-time movement model with a dynamic behavioral parameter similar to that of Eisaguirre et al. [40]. The movement process for the *j*th individual is given by:

1 $${\text{x}}_{j,i} |{\text{x}}_{j,i - 1} ,{\text{x}}_{j,i - 2} \sim \mathcal{N}\left( {{\text{x}}_{j,i - 1} + {\upgamma }_{j,i} \frac{{\Delta t_{j,i} }}{{\Delta t_{j,i - 1} }}\left( {{\text{x}}_{j,i - 1} - {\text{x}}_{j,i - 2} } \right),\;\;\Delta t_{j,i} \sigma_{x,j}^{2} {\mathbf{I}}} \right),$$

where **x**_j,i_ is a cartesian coordinate vector of the individual’s location at time t_j,i_ and **I** is the identity matrix**.** The model estimates a continuous, time-varying latent variable γ_*j,i*_ ∈ [0,1]. Higher values of γ_*j,i*_ indicate persistent, directional movements and reduced values indicate tortuous, encamped movements [40, 42, 51, 52]. We can therefore interpret higher estimated values of γ_*j,i*_ as an indicator of the degree of migratory behavior expressed along the trajectory. Within the model, γ_*j,i*_ is specified as a linear combination of environmental covariates associated with each location:

2 $${\text{logit}}(\upgamma _{j,i} {)} \sim \mathcal{N}\left( {{\mathbf{Z}}_{j,i}^{T} {\varvec{\beta}}_{j} ,\Delta t_{j,i}^{2} \sigma_{\nu ,j}^{2} } \right),$$

representing the effect of the environment on the animal’s movement pattern. Here, **Z**_*j,i*_ is a vector that contains the environmental covariates associated with each **x**_*j,i*_, and ***β***_*j*_ is a vector that weights the effects of those covariates on γ_*j,i*_. Full model statement and details are provided in Additional File 2.

We estimated individual model parameters in a Bayesian framework with Hamiltonian Monte Carlo (HMC) using Stan version 2.19.1 [53], program R version 3.6.2 [54], and the package “RStan” version 2.19.3 [55]. The model was fit to each individual season with 3 chains of 100,000 HMC iterations, including 50,000 for burn-in, and thinned by 10 (see Additional File 3 for implementation). Since the initial stage of our analysis was based at the individual level, we scaled our inference up to the annual population level with recursive Bayesian computation using a second stage Markov chain Monte Carlo (MCMC) algorithm [43, 56–58]. We modeled the population-level coefficient *β*_p,k_ for the kth covariate as:

3 $$\beta_{j,k} \sim \mathcal{N}\left( {\beta_{p,k} ,\sigma_{\beta ,k}^{2} } \right).$$

We assigned an informative prior centered on zero to each *β*_p,k_ to ensure that any apparent effects of environmental covariates detected were relatively strong (see full model statement in Additional File 2). To ensure that our results were not heavily weighted by over-representing winter movements in the dataset (that is, movements that were made after migrations had ended), we repeated the analysis and fit models to a truncated movement time-series (August 15 to November 15) and compared these to the original results.

### Environmental data

We attributed environmental variables to the caribou location data using the track annotation service Env-DATA [59] on Movebank (www.movebank.org). For each location, we obtained time-specific point estimates for air temperature (°C), snow accumulation (meters), wind speed (m sec^−1^), and standardized atmospheric pressure (Pa). These were derived from the North American Regional Reanalysis [60] and are produced at 3-h intervals and 0.3 degree spatial resolution. We also included an index of vegetation greenness, the Normalized Difference Vegetation Index (NDVI), as measured from the MODIS satellite platform for each location with a 250-m resolution [61]. NDVI was derived from 16-day composites and the best image within that time span was used as the value. We set NDVI values to 0 for all locations which had measured snow accumulation, because changes in snow cover drives a large part of the seasonal NDVI patterns in Arctic environments [62]. Bilinear interpolation was used for all attributes in Env-DATA and weather reanalysis data have been found to have good agreement with weather station data collected in the area of the herd [63, 64]. Correlations between environmental variables were all less than 0.7 and all variables were standardized to mean zero and unit variance prior to fitting the model.

To test our hypothesis that migratory behavior is a function of experienced environmental covariates, we fit one model that included the main effects for each environmental covariate. We included an interaction between temperature and snow depth to test for potential additional effects of snow at a given temperature [28]. The expected value of movement persistence was thus modeled as:

4 $$\begin{aligned} E({\text{logit}}(\upgamma_{j,i} {)}) & = {\varvec{Z}}_{j,i}^{T} {\varvec{\beta}}_{j} = \beta_{0} + \beta_{1} \cdot {\text{Temp(}}x_{j,i} {)} + \beta_{2} \cdot {\text{Snow(}}x_{j,i} {)} \\ & \quad + \beta_{3} \cdot {\text{Temp(}}x_{j,i} {)} \cdot {\text{Snow(}}x_{j,i} {)} + \beta_{4} \cdot {\text{Wind(}}x_{j,i} {)} \\ & \quad + \beta_{5} \cdot {\text{NDVI(}}x_{j,i} {)} + \beta_{6} \cdot {\text{Pressure(}}x_{j,i} {)}{\text{.}} \\ \end{aligned}$$

We interpreted multiple years of 90% credible intervals that did not overlap zero for each year of the population-level model to indicate effects of the environmental covariate [65, 66]. To visualize our results at the landscape scale, we downloaded environmental rasters of these given covariates at three characteristic periods (early-, middle-, and late-autumn) in 2010. We then mapped expected movement patterns over the landscape using the equation $\widehat{{\gamma }_{\text{i}}}={logit}^{-1}({{{\varvec{Z}}}_{i}}^{{\varvec{T}}}{\widehat{{\varvec{\beta}}}}_{p})$, where $\hat{\user2{\beta }}_{p}$ represents the posterior mean for the population-level coefficients and ${{{\varvec{Z}}}_{i}}^{{\varvec{T}}}$ the vector of observed environmental conditions in each pixel at a representative date and time. Stan and R code used to implement our approach is provided in Additional File 4.

## Results

**Fig. 1:**
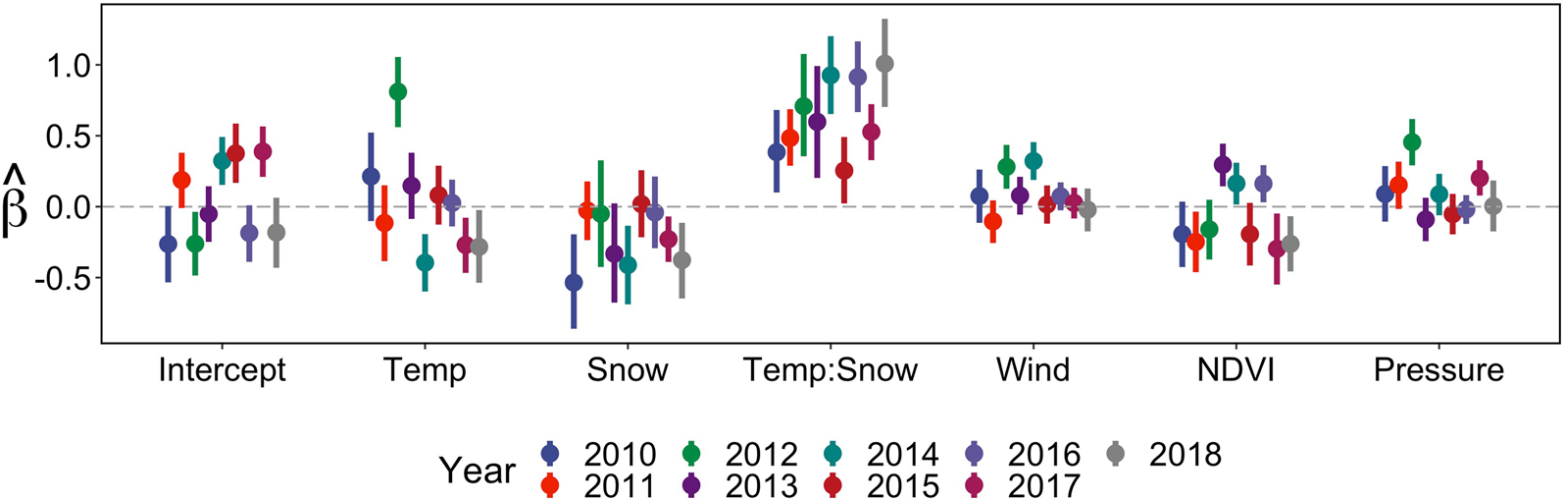
Annual estimated population-level coefficients (points) and 90% credible intervals (bars) for the effect of environmental variables on migratory movements ($\widehat{{\gamma }_{\text{i}}}$) from the dynamic-parameter correlated random walk model. Environmental variables were standardized and the model was fitted to individual tracks of caribou data from the Western Arctic Herd, Alaska, 2010–2018

Across all 9 years, the combination of snow and temperature had the strongest influence on autumn migratory movements, with estimated coefficients of the interaction term and 90% credible intervals (CI) that were consistently above zero (Fig. 1). Chain mixing and potential scale reduction statistics ($\widehat{R}$) less than 1.01 for all 389 individual season models indicated convergence to the posterior distribution [53].

**Fig. 2:**
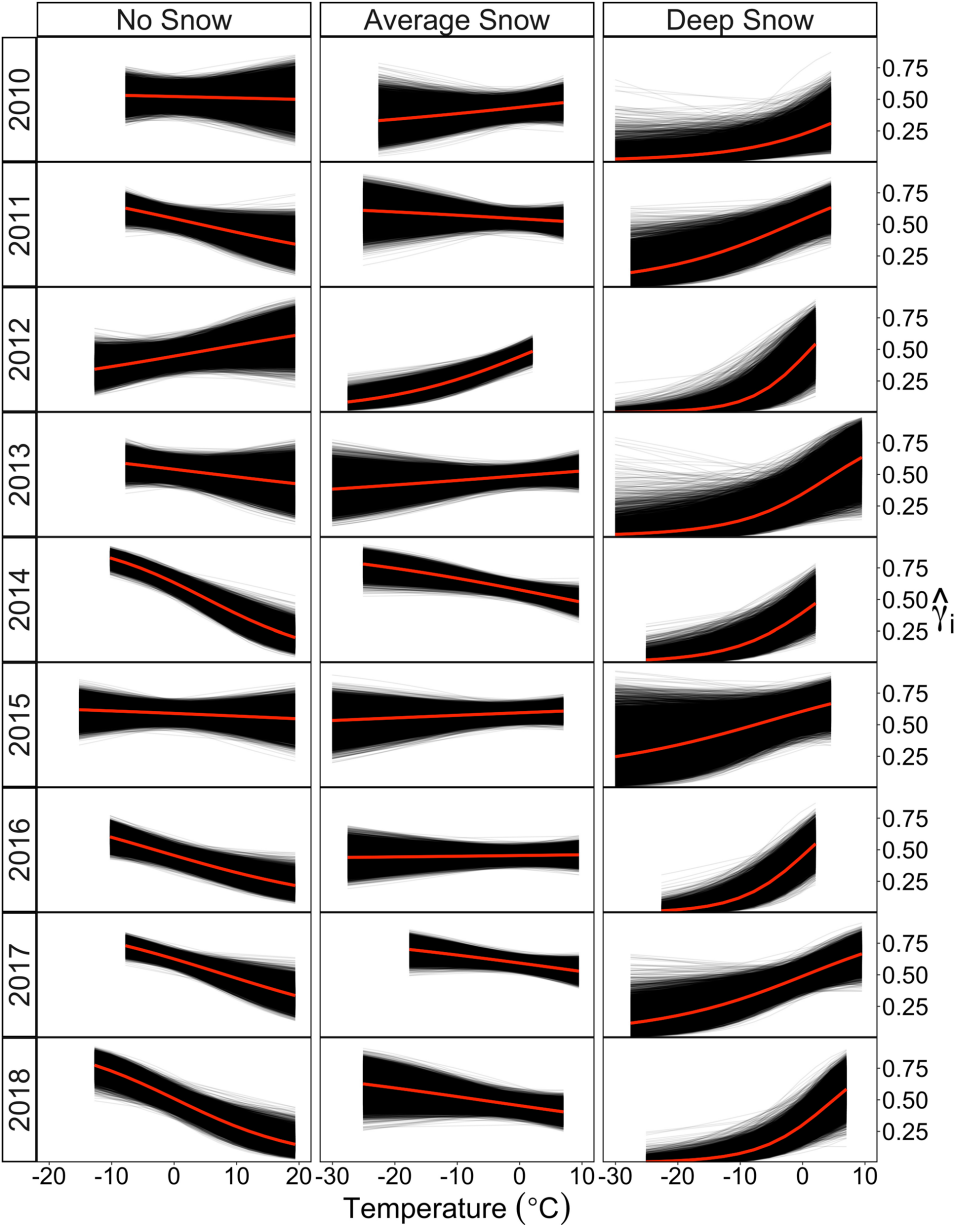
The effect of temperature at three different snow depth levels for each year from the population-level fit of the dynamic-parameter correlated random walk movement model fitted to caribou location data of the Western Arctic Herd, Alaska, 2010–2018. For each year, the predicted effect of temperature (x-axis) on the movement parameter ($\widehat{{\gamma }_{\text{i}}}$; y-axis) is plotted across 3 levels of snow depth (no snow = 0 cm, average snow = 11 cm, and deep snow = 46 cm). Each black curve is given by the equation ${\widehat{{\gamma }_{\text{i}}}}^{(l)}={logit}^{-1}({{{\varvec{Z}}}_{{\varvec{i}}}}^{{\varvec{T}}}{{\widehat{{\varvec{\beta}}}}_{{\varvec{p}}}}^{\left({\varvec{l}}\right)})$ for the *l*th Markov-Chain Monte Carlo iteration (termed posterior realizations), and the red line indicates the mean. Annual plots are cut off to the observed range of values for each year

**Fig. 3:**
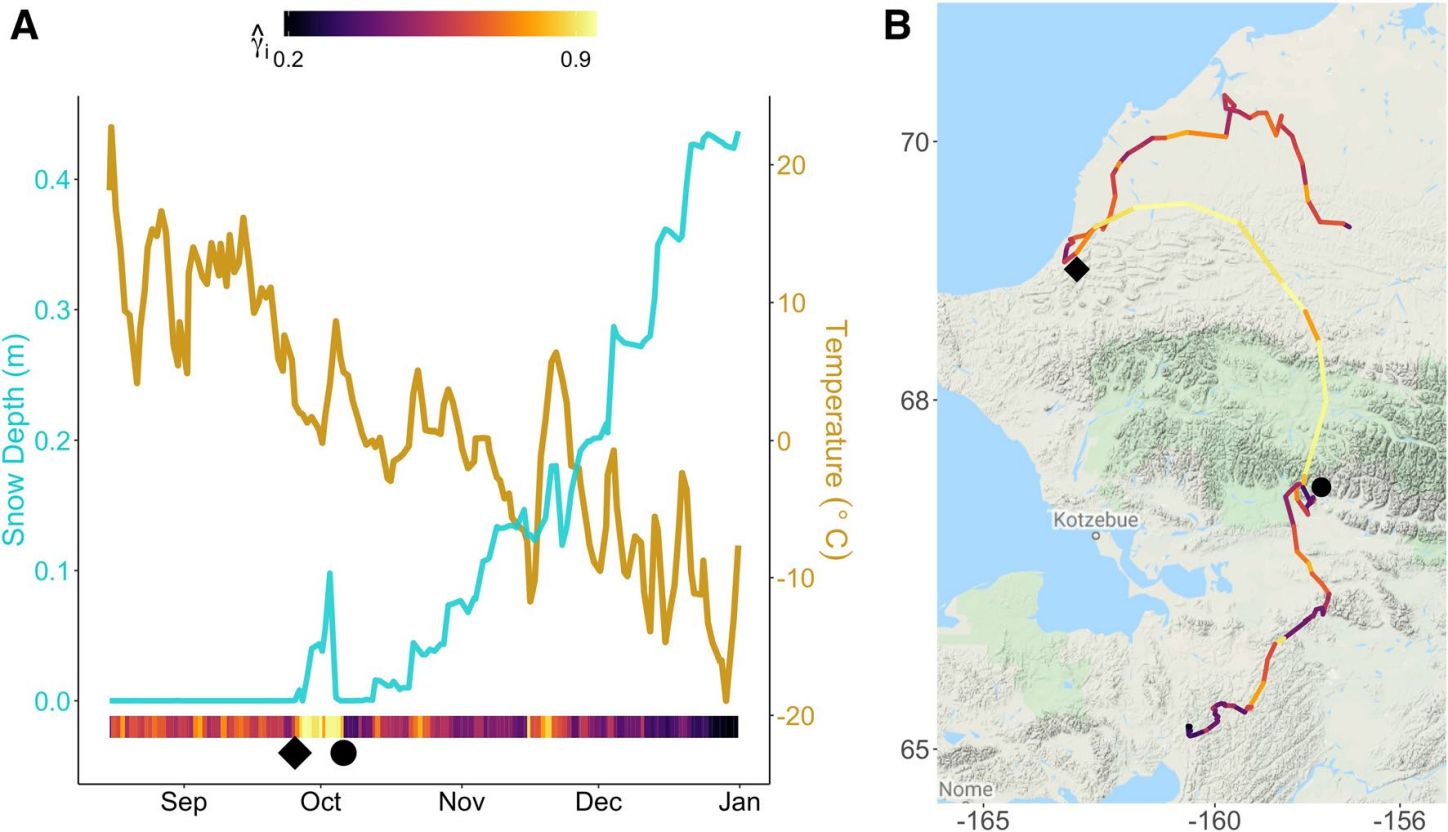
Environmental conditions experienced by an individual caribou of the Western Arctic Herd (**A**) and movement track from Aug 15 to Jan 1, 2010 (**B**). Panel **A** indicates the snow depth (blue) and temperature (gold) at each location as extrapolated from the North American Reanalysis Model (National Centers for Environmental Prediction 2005). Estimated migratory movement ($\widehat{{\gamma }_{\text{i}}}$) for the individual is illustrated in the bottom bar from dark blue (low persistence and localized movements) to yellow (high persistence and directional movements). Panel **B** illustrates the measured caribou movements for the same time period and are colored by the same color scheme for migratory movement as in panel **A**. The diamond and circle indicate the start and end, respectively, of the longest migratory movement in both panels

Interpretation of the main effects of temperature and snow on migratory movement was nuanced due to the consistently significant interaction term (Fig. 1, Additional file 1: Table S2) and was best interpreted through comparing the effect to migratory movement across a range of both temperature and snow values (Fig. 2). When snow was absent, decreasing temperatures alone resulted in increased migratory movement for all but one year, suggested by higher γ_*j,i*_ at low temperatures (Fig. 2). Accumulating snow depth modulated this relationship between temperature and migratory movement, such that snow accumulation at relatively warmer temperatures in autumn resulted in higher γ_*j,i*_. This was pronounced for the first snow event and early accumulation of snow depth, which were consistently associated with elevated γ_*j,i*_, such that individuals typically exhibited more persistent movements within 10 days of early season snow events (Fig. 3; Additional File 1: Figure S1). Notably, migratory movements were clearly altered as environmental conditions moderated in the days following such events, and animals often exhibited more localized, slower movements (decreased γ_*j,i*_) after reaching snow-free areas farther south (e.g., Fig. 3; Additional File 1: Figure S2). Snow appeared to become a hindrance to movement as it accumulated, such that deep snow (e.g., more than 40 cm) and cold temperatures (such as − 20 to – 30 °C) were associated with the most encamped movement behaviors (Figs. 2, 3). For 8 of 9 years, the relationship between temperature and γ_*j,i*_ inverted at an average depth of 12 cm (range 2 [2010]–21 cm [2017]).

Wind, NDVI, and air pressure had less pronounced and more variable effects on migratory movements. Windy conditions were generally associated with increased migratory movements, as the estimated coefficient was positive for 7 out of 9 years (2010 and 2012–2017). However, evidence was weak as of those, only two had 90% CIs that did not include zero (Fig. 1; Additional File 1: Figure S3). Increased migratory movements were generally associated with decreasing NDVI values, with negative coefficients in 6 years (90% CI below zero for 3 years; Fig. 1; Additional File 1: Figure S3). Barometric pressure exhibited a generally positive but again weak effect on migratory movements, with 6 years of positive coefficients and of those, two with a 90% CI that did not include 0 (Fig. 1; Additional File 1: Figure S3). These patterns among environmental variables and migratory movement were consistent, albeit less pronounced, when models were fitted to the truncated timeseries data that ended Nov. 15 (Additional File 1: Figure S4 & Figure S5).

**Fig. 4:**
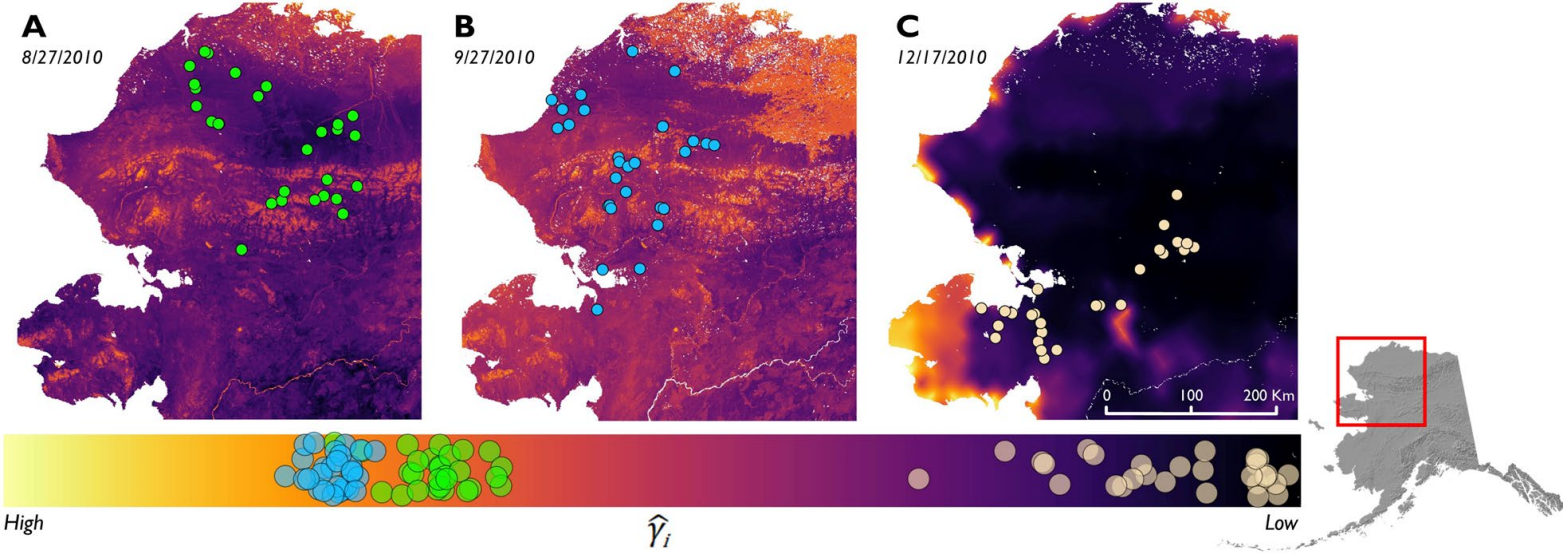
Predicted migratory movement ($\widehat{{\gamma }_{\text{i}}}$) for the range of the Western Arctic Herd given the environmental conditions at three time periods − 8/27 (**A**), 9/27 (**B**), and 12/17 (**C**) − and corresponding population-level model results for individuals in 2010. Dark colors indicate reduced movement persistence (low $\widehat{{\gamma }_{\text{i}}}$) and yellow indicate persistent movement (high $\widehat{{\gamma }_{\text{i}}}$). Caribou locations are displayed in each panel and colored by day, and the legend bar along the bottom indicates the range of $\widehat{{\gamma }_{\text{i}}}$ and the corresponding values at the observed caribou locations

When visualizing the spatiotemporally explicit movement patterns predicted from our model, the result was extremely heterogenous expected migratory movements that were highly dependent upon where animals were located on the landscape and were temporally dynamic (Fig. 4). The degree of expected migratory movement at a given time and place was a function of the entire suite of environmental factors experienced by individuals. Once snow depth increased to mid-winter depths, movements were predicted to become encamped and homogeneous in the majority of the range regardless of how far south individuals were (Fig. 4) as individuals ceased migration and entered an overwinter movement regime.

## Discussion

More than a century after Dugmore [32] postulated that accumulating snow, decreasing temperatures, and changes in weather affected autumn migration in caribou, we used modern technology and statistical approaches to quantify the dynamic response by caribou to localized snow and temperature conditions and determined that migration is continuously reassessed throughout the migratory period. Whereas the response to these variables scaled to a consistent population-level pattern (Figs. 1, 2), the dispersion of individuals across a wide geographic area resulted in notable variation in migratory behavior among individuals for any given time due to differences in conditions across the region (Fig. 4). Our findings suggest that autumn migration can be envisioned as the recently introduced concept of migratory pacing, in which individuals continuously adjust migratory behavior based on experienced in situ environmental conditions [40], rather than a single discrete action (i.e. “on/off”). Migratory pacing can include stopover behavior and unifies a set of ideas describing migration patterns across the spring and autumn legs of the complete migratory cycle. While many temperate migrants pace spring migration with the flush of resource quality across the landscape (i.e. ‘green wave surfing’) [6–10], senescence in autumn is a gradual and protracted decline of vegetation quality that largely ends when snow accumulates [62]. In contrast to spring migration, our results indicate that migrants largely pace autumn migration with respect to indicators of the approaching winter, similar to the ‘frost wave’ suggested by Xu and Si [67]. For caribou, these findings are congruent with early field observations which speculated such a mechanism [32, 34, 35].

Our findings have two important implications for the migratory patterns of populations. The first is that experienced environmental conditions across a population prior to and during migration may exhibit a wide range depending on the spatial distribution of individuals. This is pronounced in caribou, as they are typically dispersed in late summer [33, 68, 69], and this is especially true for the Western Arctic Herd [44, 46] (Fig. 4). Secondly, individuals respond to proximate environmental cues in a common manner despite this widespread spatial dispersion of groups. This finding is similar to that for golden eagles (*Aquila chrysaetos*), which use thermal uplift as a flight subsidy along a variety of autumn (and spring) migration routes [40], as well as elk herds in the Greater Yellowstone Ecosystem, which rely on similar environmental cues for migration timing despite ranges being spatially distinct [70]. Our findings indicate that caribou generally exhibit a common behavioral response to similar proximate weather conditions (snow and temperature) they experience. One notable commonality was elevated movement persistence after the first snowfall event of the season (such as the individual depicted in Fig. 3), which appeared to be representative of the general response we found in our population-level results. The dispersed distribution of the herd in late summer results in individuals experiencing different environmental conditions which, in turn, leads to different individual-level migratory decisions. These then scale up to the observed variability and asynchrony in migratory patterns (this study) observed at the population level [44, 46]. More generally, the consistent population-level responses to environmental cues that we detected suggests that variability in environmental conditions experienced across a population’s distribution in a given year can explain why autumn migrations can exhibit such wide variability in timing across many taxa [22, 23, 71].

Assessing the influence of the environment along the entire migration trajectory provides a mechanistic link between broad-scale weather patterns and migration, suggesting that changes in the prevailing climate may result in changes to migratory patterns. In the range of the Western Arctic Herd, the climatic trend has been for warmer autumns [72] and has coincided with progressively later autumn migrations over multiple decades [44, 46, 73]. Shifts in autumn migration timing have been linked to environmental trends in other species as well, such as for Chukchi Sea Beluga whales (*Delphinapterus leucas*) that now migrate later as seasonal sea-ice formation has become delayed [74]. Timing of elk autumn migration in the Greater Yellowstone Ecosystem was found to be highly plastic from 2001 to 2017 and corresponded to changes in snow patterns for many of the herds [70]. Understanding how climate influences migration behavior is important for predicting how long-distance migrant populations may or may not respond adaptively to future climate change [75]. This is especially pertinent for rural Arctic subsistence communities, whose cultural identity and way of life date back more than 10,000 years and rely on harvesting caribou during migration [76]. Given the rapid changes currently being observed in the Arctic and even greater ones predicted with climate change [77, 78], our results indicate that caribou migrating long-distances, and perhaps other long-distance migrants, are highly plastic in their decision of when and at what pace to migrate, and that further migration delays could occur if the warming trend continues.

In the later part of the season we analyzed (i.e., late November–December), we found that movement persistence consistently reached its lowest levels as winter conditions set in, which were characterized by deep snow and cold temperatures. This aligns with documented increased costs of winter movements, given that the energy expenditure to move through snow increases exponentially with increased sinking depth (and thus snow depth) [79]. Winter movement rates progressively diminish throughout winter [50] and concurrently, metabolic rates and energy requirements in caribou decrease [80]. These are some of the numerous adaptations by northern species to survive the long winter months [81] and highlight the importance of incorporating snow metrics in studies of animal movements in northern ecosystems [82, 83].

Our primary finding that migratory movements are a response to dynamic and localized temperature and snow patterns is consistent with previous research for species exhibiting shorter and less demanding migrations, such as mule deer [23, 29, 30], white-tailed deer [28, 84], elk [70], and red deer [27]. We also found evidence that vegetation and meteorological conditions can influence migratory movements to lesser degrees. For most years observed, we found a negative relationship between NDVI and migratory movements, indicating caribou travelled more persistently as vegetation senesced and, conversely, were more localized when animals encountered greener vegetation (Fig. 1; Additional File 1: Figure S3). This relationship was not likely to have been driven by snow accumulation (when NDVI values became zero) given the variability in response across years we observed. Our finding that autumn migration timing is related to fall senescence is similar to European populations of roe and red deer, for which migration timing is linked to decreasing plant productivity, as measured by NDVI [26]. Under the migratory pacing concept, individuals may delay not only the start of autumn migrations but also slow down or pause migration *en route* (stopover) if they encounter improved foraging conditions. This is similar to mule deer, for which stopover sites have higher NDVI values than neighboring migration corridors [38]. Such a tactic could prolong access to good foraging conditions before winter sets in and reduce competition on winter areas by delaying arrival as long as possible. We also found that migratory movements were more persistent on windy, high pressure days in some years, suggesting that migration speed may be modulated by fair weather conditions. Other, less predictable meteorological conditions not considered here, such as rain on snow events, are known to influence caribou movements as well [85]. Autumn migration has been observed during warm weather in the past [47], similar to an anomalous year in our data, and this highlights the need for further research into autumn migration.

## Conclusions

By treating migration as a series of directional and persistent migratory movements and classifying these as a continuous metric, we show that decreasing temperature and increasing snow depth influence when and how caribou migrate in autumn. These quantitative findings align with the early observations of naturalists and field biologists that have accrued over the last century. Because individuals of this caribou herd are dispersed across a large spatial extent in autumn, variability in experienced conditions results in a wide range of observed migration patterns. This mechanism of pacing autumn migration based on indicators of the approaching winter is analogous to the more widely researched mechanism of spring migration, when many migrants pace migration with a resource wave, and highlights the different environmental stimuli migrants have adapted to respond to throughout their annual cycle.

## Supplementary Information

**Additional file 1**. Additional figures and tables.

**Additional file 2**. Stan model details.

**Additional file 3**. Individual-level model implementation.

**Additional file 4**. Population-level model implementation.

## Data Availability

The GPS data used in this study are owned by National Park Service and Alaska Department of Fish and Game, both of which restrict the availability of location data for a harvestable species. All GPS data are stored in the public repository IRMA and are available from the project leads on reasonable request: https://irma.nps.gov/DataStore/Reference/Profile/2260262.
