# Supplementary material for: Mechanistic movement models identify continuously updated autumn migration cues in Arctic caribou
Source: Mov Ecol. 2021 Nov 1;9:54. doi: 10.1186/s40462-021-00288-0 (PMC8559358; doi:10.1186/s40462-021-00288-0)
Supplement: Supplementary file 1 — Additional file 1. Additional figures and tables. [file 40462_2021_288_MOESM1_ESM.docx]

**Additional File 1 – Figures and Tables**

Cameron, MD, JM Eisaguirre, GA Breed, K Joly, & K Kielland. 2021. Mechanistic movement models identify continuously updated autumn migration cues in Arctic caribou. Movement Ecology. DOI: 10.1186/s40462-021-00288-0.

Table S1: Number of individual seasons in each year of analysis for autumn migration of Western Arctic Herd caribou, Alaska.

| Year | Individual Seasons |
| --- | --- |
| 2010 | 28 |
| 2011 | 38 |
| 2012 | 33 |
| 2013 | 35 |
| 2014 | 42 |
| 2015 | 42 |
| 2016 | 66 |
| 2017 | 62 |
| 2018 | 43 |

Table S2: Probabilities of each parameter estimate being different from zero (either positive or negative coefficient) from the fitted correlated random walk movement model for caribou data of the Western Arctic Herd, Alaska. Probabilities estimated by applying Monte Carlo integration to the marginal posteriors of the population-level parameters

|  | Probability of $\beta$ estimates differing from zero | | | | | | | | |
| --- | --- | --- | --- | --- | --- | --- | --- | --- | --- |
| Parameter | 2010 | 2011 | 2012 | 2013 | 2014 | 2015 | 2016 | 2017 | 2018 |
| Intercept | 0.95 | 0.94 | 0.97 | 0.67 | 1.00 | 1.00 | 0.94 | 1.00 | 0.89 |
| Temp | 0.87 | 0.76 | 1.00 | 0.85 | 1.00 | 0.74 | 0.60 | 0.99 | 0.96 |
| Snow | 0.99 | 0.58 | 0.59 | 0.94 | 0.99 | 0.55 | 0.61 | 0.99 | 0.99 |
| Temp x Snow | 0.99 | 1.00 | 1.00 | 1.00 | 1.00 | 0.97 | 1.00 | 1.00 | 1.00 |
| Wind | 0.74 | 0.88 | 1.00 | 0.83 | 1.00 | 0.57 | 0.89 | 0.65 | 0.59 |
| NDVI | 0.92 | 0.97 | 0.89 | 1.00 | 0.97 | 0.93 | 0.98 | 0.98 | 0.99 |
| Pressure | 0.78 | 0.93 | 1.00 | 0.84 | 0.84 | 0.73 | 0.62 | 1.00 | 0.51 |

**
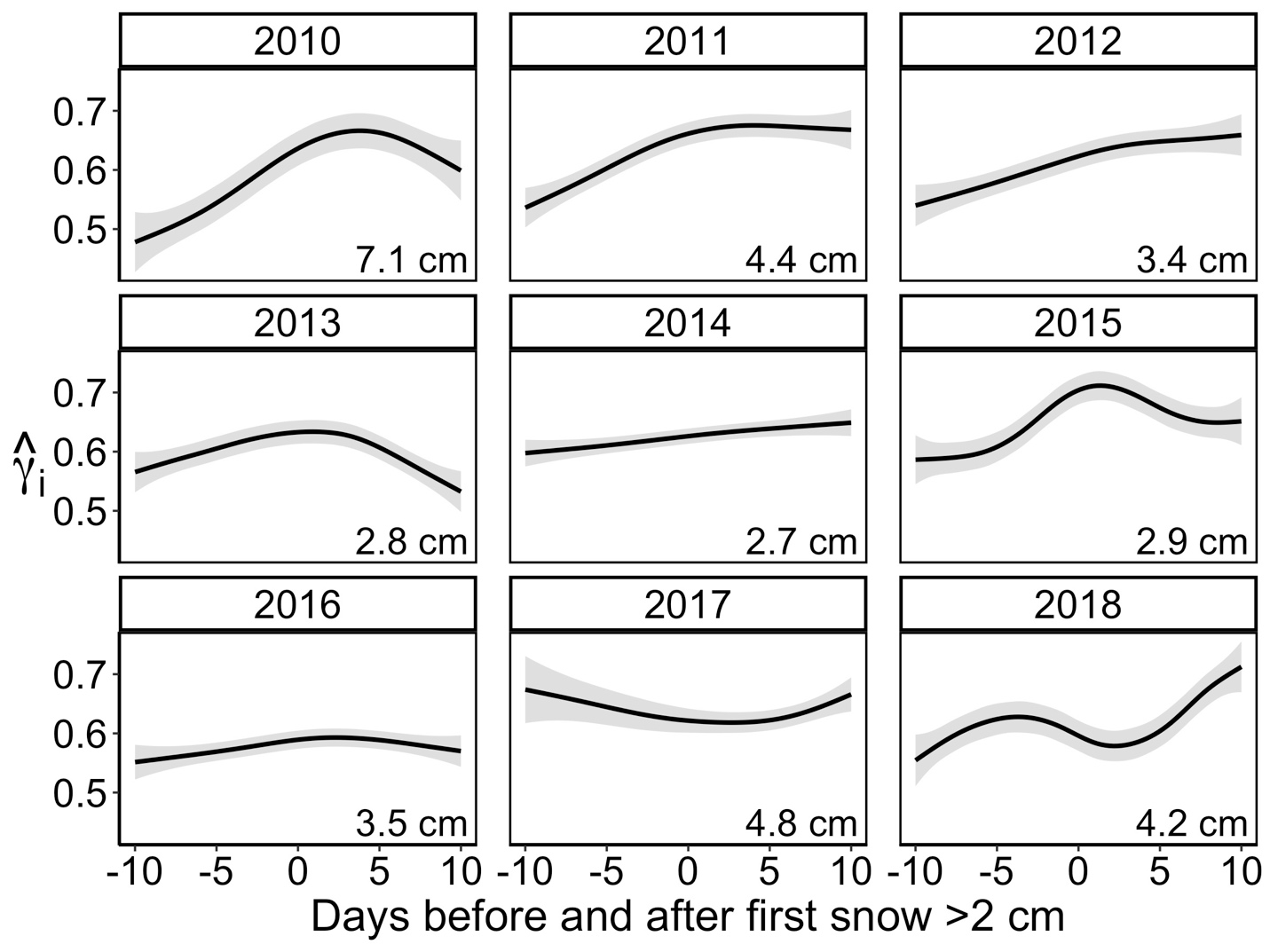
**

Figure S1: Fitted $\hat{}_{\text{i}}$ (y-axis) by year (black lines) smoothed across individuals in relation to the 10 days preceding and following the first appreciable snowfall (> 2 cm) experienced by individual collared caribou of the Western Arctic Herd, Alaska, from 2010-2018. Fitted lines and 95% confidence intervals were generated from a generalized additive model fit with a cubic regression spline for the smoothing parameter for each year using the posterior means of the correlated random walk movement model. Snow data was obtained from the National Centers for Environmental Prediction (2005) and are specific to the time and place of the caribou GPS location. The average snow depth (cm) on day zero for each year is presented in the bottom right of each plot. In general, caribou exhibited elevated $\hat{}_{\text{i}}$ values (more persistent migratory movement) after the first appreciable snowfall, although this relationship was complicated by the temperature, realized snow depth after the storm, and caribou movement in the days following the event.

Figure S2: Environmental conditions experienced by an individual caribou of the Western Arctic Herd (A) and movement track from Aug 15 – Jan 1, 2018 (B). Panel A indicates the snow depth (blue) and temperature (gold) at each location as extrapolated from the North American Reanalysis Model (National Centers for Environmental Prediction 2005). Estimated migratory movement ($\hat{{}_{\text{i}}}$) for the individual is illustrated in the bottom bar from dark blue (low persistence and localized movements) to yellow (high persistence and directional movements). Panel B illustrates the measured caribou movements for the same time period and are colored by the same color scheme for migratory movement as in panel A.


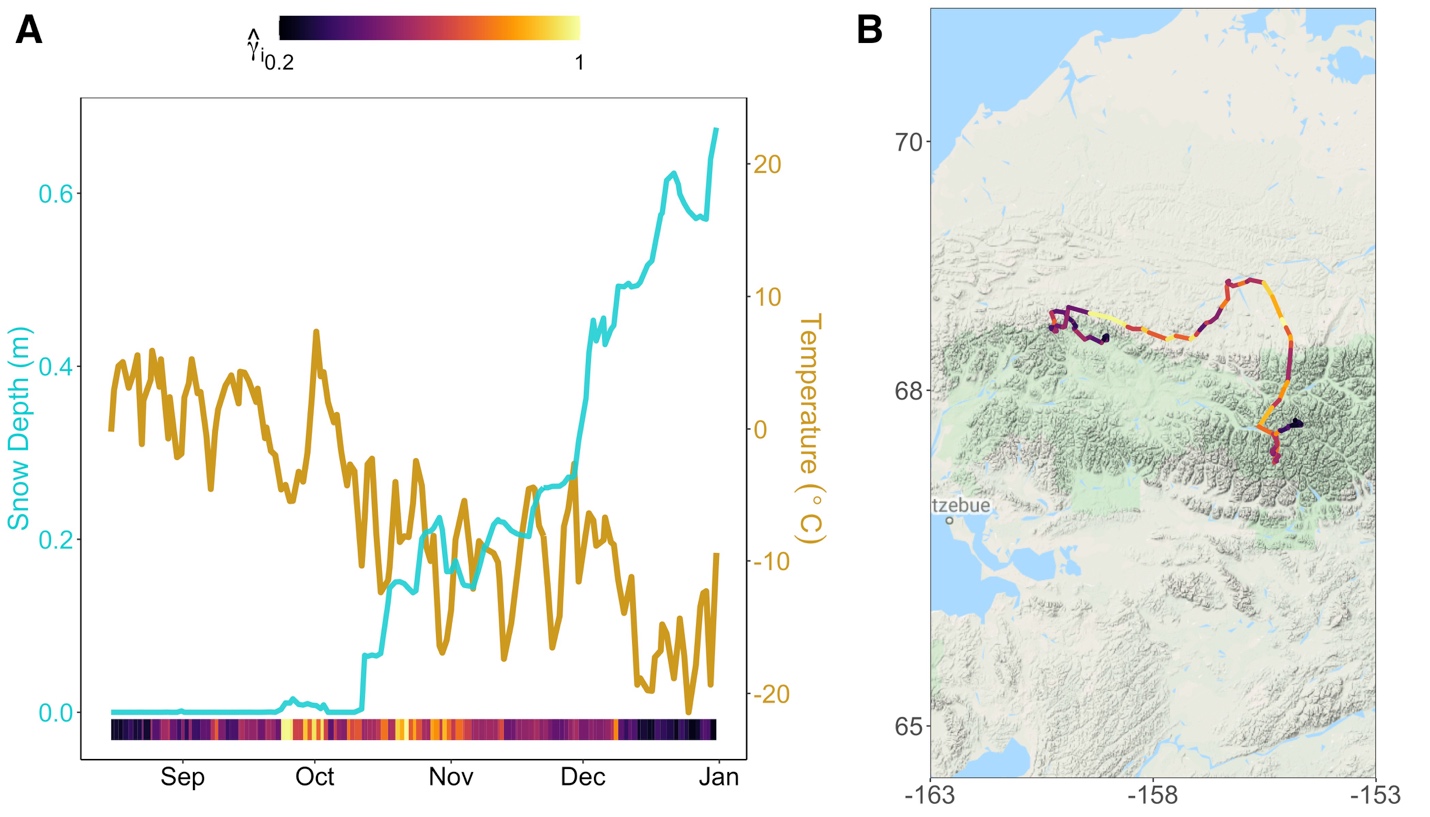


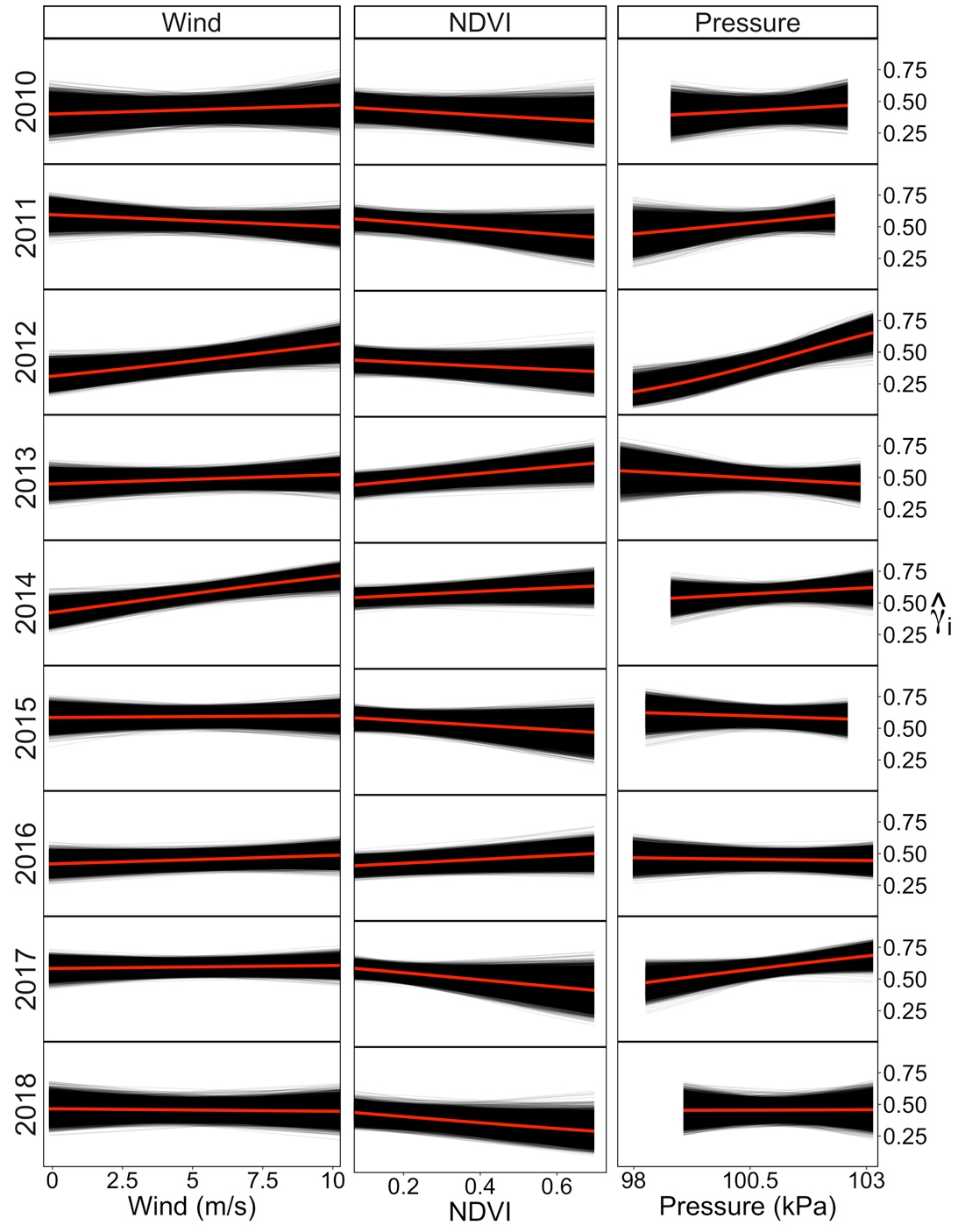


Figure S3: The effect of wind, NDVI, and standardized air pressure for each year from the population-level fit of the dynamic-parameter correlated random walk movement model fit to caribou location data of the Western Arctic Herd, Alaska, 2010-2018. For each year, the predicted effect of the respective covariate (x-axis) on the movement parameter ($\hat{{}_{\text{i}}}$; y-axis) is plotted. Each black curve is given by the equation ${\hat{{}_{\text{i}}}}^{(k)}={logit}^{-1}({\boldsymbol{Z}_{\boldsymbol{i}}}^{\boldsymbol{T}}{{\hat{\boldsymbol{\beta}}}_{\boldsymbol{p}}}^{\left( \boldsymbol{k} \right)})$ for the kth Markov-Chain Monte Carlo iteration (termed posterior realizations), and the red line indicates the mean.


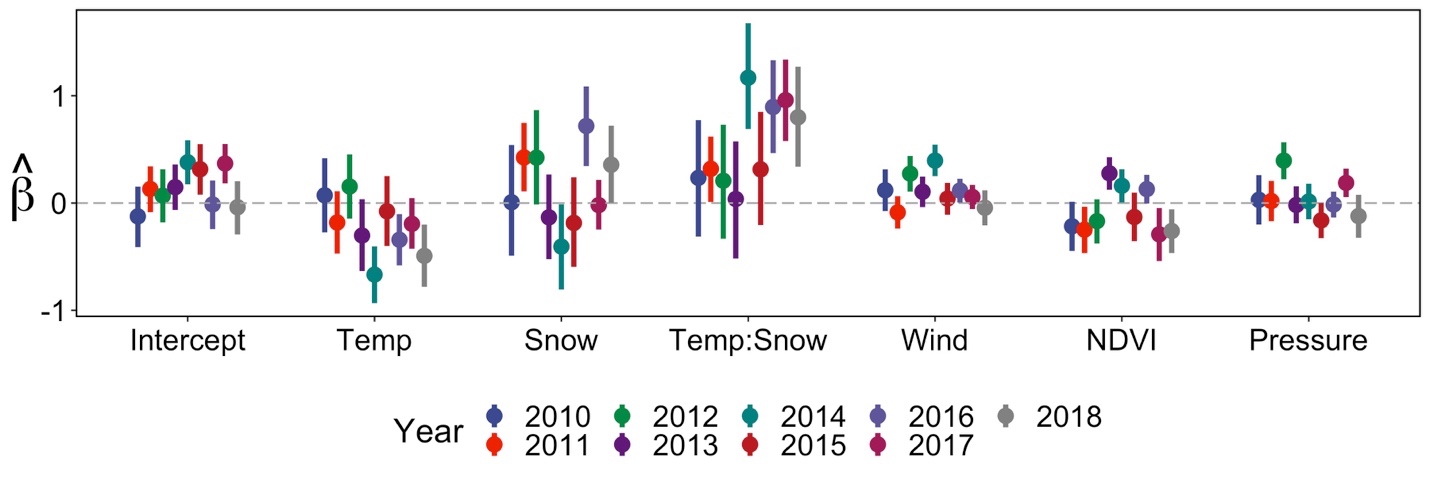


Figure S4: Annual estimated population-level coefficients (points) and 95% Credible Intervals (bars) for the effect of environmental variables on migratory movements ($\hat{{}_{\text{i}}}$) from the dynamic-parameter correlated random walk model. Model was fit to individual tracks of caribou data from the Western Arctic Herd, Alaska, 2010-2018 and the timeseries was restricted to data spanning August 15 – November 15 (rather than the August 15 – December 31 used in Figure 1) to assess the impact of the end of the migratory period.


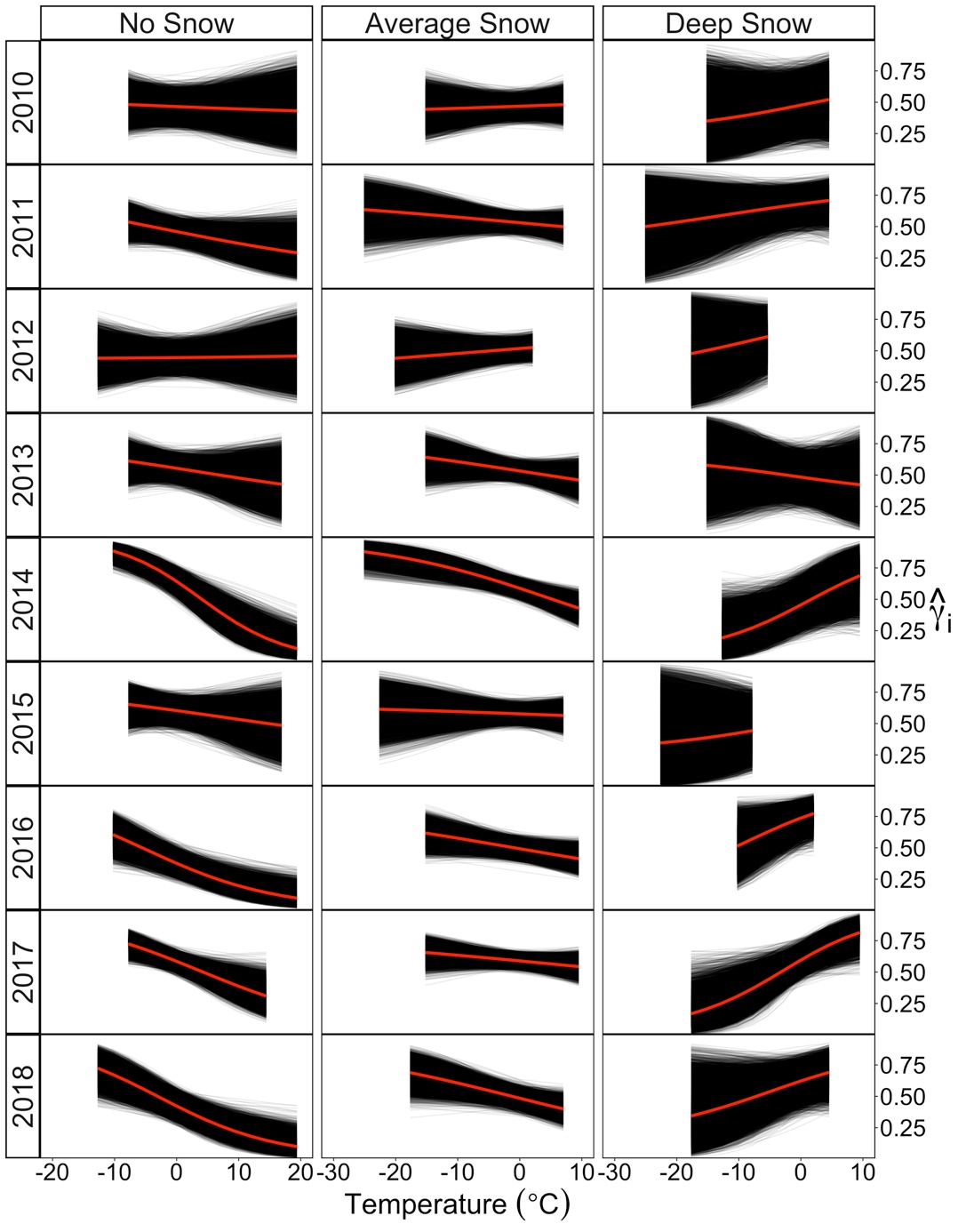


Figure S5: The effect of temperature at three different snow depth levels for each year from the population-level fit of the dynamic-parameter correlated random walk movement model fit to caribou location data of the Western Arctic Herd, Alaska, 2010-2018. For each year, the predicted effect of temperature (x-axis) on the movement parameter ($\hat{{}_{\text{i}}}$; y-axis) is plotted across 3 levels of snow depth (no snow = 0 cm, average snow = 11 cm, and deep snow = 37 cm). Each black curve is given by the equation ${\hat{{}_{\text{i}}}}^{(k)}={logit}^{-1}({\boldsymbol{Z}_{\boldsymbol{i}}}^{\boldsymbol{T}}{{\hat{\boldsymbol{\beta}}}_{\boldsymbol{p}}}^{\left( \boldsymbol{k} \right)})$ for the kth Markov-Chain Monte Carlo iteration (termed posterior realizations), and the red line indicates the mean. Annual plots are cut off to the observed range of values for each year. The timeseries considered was reduced to data from August 15 to November 15 each year (rather than the August 15 – December 31 used in Figure 1) to assess the impact of the end of the migratory period.
