# Supplementary material for: Mechanistic movement models identify continuously updated autumn migration cues in Arctic caribou
Source: Mov Ecol. 2021 Nov 1;9:54. doi: 10.1186/s40462-021-00288-0 (PMC8559358; doi:10.1186/s40462-021-00288-0)
Supplement: Supplementary file 2 — Additional file 2. Stan model details. [file 40462_2021_288_MOESM2_ESM.docx]

**Additional File 2 – Stan model details**

Cameron, MD, JM Eisaguirre, GA Breed, K Joly, & K Kielland. 2021. Mechanistic movement models identify continuously updated autumn migration cues in Arctic caribou. Movement Ecology. DOI: 10.1186/s40462-021-00288-0.

Full model statement for the hierarchical, continuous-time movement model with a dynamic behavioral parameter:


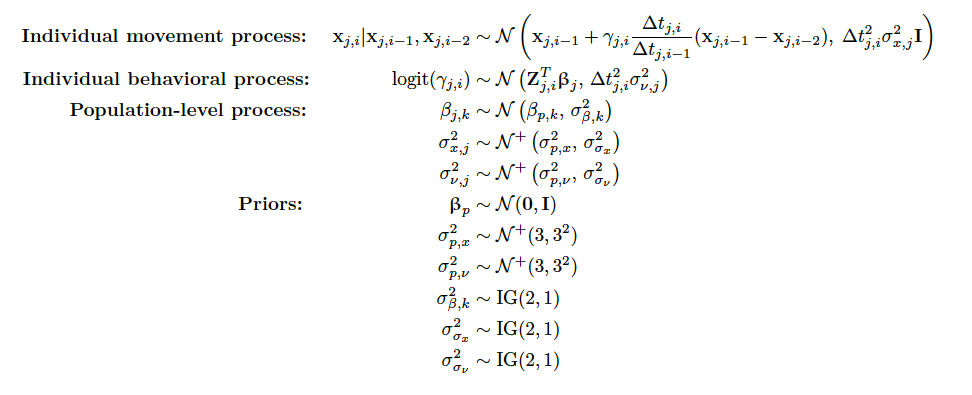


Stan code for the continuous time, dynamic parameter correlated random walk movement model. Corresponding Stan-type file available from co-authors upon request.

data {

int N; // # of fixes in track

vector[N] x; // x coordinates

vector[N] y; // y coordinates

vector[N] dt; // time intervals

vector[N] cov_1; // covariates

vector[N] cov_2;

vector[N] cov_3;

vector[N] cov_4;

vector[N] cov_5;

}

parameters {

vector[N] gamma_raw; // logit behavior parameter -- time-varying, correlates steps

real<lower=0> sigmax; // movement process noise in x

real<lower=0> sigmav; // behavior process noise

vector[7] beta; // covariate coefficients

}

transformed parameters{

// Introduce the logit link on the behavior parameter

vector<lower=0,upper=1>[N] gamma;

for(j in 1:N){

gamma[j] = inv_logit( gamma_raw[j] );

}

}

model {

// Prior on behavior process noise -- assume there is variability in behavior

sigmav ~ inv_gamma( 2 , 5 );

// Priors on movement process noise -- close to zero

sigmax ~ inv_gamma( 3 , 1 );

// Priors on coefficients -- null model is no effect of covariates on behavior

beta ~ normal( 0 , 1 );

for (i in 3:N) {

// Behavior is a linear combination of covariates

gamma_raw[i] ~ normal( beta[1] + beta[2] * cov_1[i] + beta[3] * cov_2[i] + beta[4] * cov_1[i]*cov_2[i] + beta[5] * cov_3[i] + beta[6] * cov_4[i] + beta[7] * cov_5[i], dt[i] * sqrt(sigmav) );

// Movement process is independent in x and y

x[i] ~ normal( x[i-1] + gamma[i] * ( dt[i] / dt[i-1] ) * ( x[i-1] - x[i-2] ) , dt[i] * sqrt(sigmax) );

y[i] ~ normal( y[i-1] + gamma[i] * ( dt[i] / dt[i-1] ) * ( y[i-1] - y[i-2] ) , dt[i] * sqrt(sigmax) );

}

}
