# Supplementary material for: Mechanistic movement models identify continuously updated autumn migration cues in Arctic caribou
Source: Mov Ecol. 2021 Nov 1;9:54. doi: 10.1186/s40462-021-00288-0 (PMC8559358; doi:10.1186/s40462-021-00288-0)
Supplement: Supplementary file 3 — Additional file 3. Individual-level model implementation. [file 40462_2021_288_MOESM3_ESM.docx]

**Additional File 3 – Individual-level model implementation**

Cameron, MD, JM Eisaguirre, GA Breed, K Joly, & K Kielland. 2021. Mechanistic movement models identify continuously updated autumn migration cues in Arctic caribou. Movement Ecology. DOI: 10.1186/s40462-021-00288-0.

Example code to implement the continuous time, dynamic parameter correlated random walk movement model. Corresponding R-type file available from co-authors upon request.

library(rstan)

library(dplyr)

library(lubridate)

wah.fall <- read.csv("Data/Data for CRW model - 2020-02-21.csv")

wah.fall$timestamp <- ymd_hms(wah.fall$timestamp)

wah.fall <- subset(wah.fall, yday(timestamp) >= 227) # Start timeseries on Aug 15

#################

## Run as a loop through all individuals

#################

## Make a file for the parameter estimates:

output=data.frame("param","mean", "se_mean","sd","x2.5.","x25.","x50.","x75.","x97.5.","n_eff","Rhat","id","year")

write.table(output,"Summary of stan models.csv",sep=",", col.names=FALSE, row.names = FALSE)

uniq.ids <- as.character(unique(wah.fall$ID))

for (i in 1:length(uniq.ids)){

this.id <- uniq.ids[i]

hold.dat <- subset(wah.fall,ID==this.id)

# Calculate dt then toss the first point since it was used to calculate dt

hold.dat$diff <- as.double(hold.dat$timestamp - dplyr::lag(hold.dat$timestamp)) # calculates the time step for points.

hold.dat <- hold.dat[-1,]

hold.dat$diff <- hold.dat$diff/8 # sets dt to equal 1 for a scheduled interval (8 hrs) and 2 for a missed fix, etc.

# Number of points in track

N = as.integer(nrow(hold.dat))

# Assign covariates to model variables

cov_1 = hold.dat$temp # vectors of length N

cov_2 = hold.dat$snow.depth

cov_3 = hold.dat$wind # vectors of length N

cov_4 = hold.dat$ndvi # vectors of length N

cov_5 = hold.dat$pressure

x=hold.dat$x.km # x coords; vector of length N

y=hold.dat$y.km # y coords

dt= hold.dat$diff # time intervals in hours; vector of length N

df=list(x=x,y=y,N=N,dt=dt, cov_1=cov_1, cov_2=cov_2, cov_3 = cov_3, cov_4 = cov_4,cov_5 = cov_5) # Saving data for model this way got around a bug when passing stan() a list

# Fit model in Bayesian framework in Stan

stan.fit = stan("R Code/crw_stan.stan",

data = df,

chains = 3,

iter = 100000,

thin=10, # thinning 3 chains of 100,000 by 10 will give you 30,000 samples to approximate posterior (adjust if needed for memory)

cores = 3,

control = list(adapt_delta = 0.9))

## Save the stanfit object

save(stan.fit,file=paste0("Output/",this.id,".RData"))

## This section will save the summary output as a csv to check things later

iter.rhat <- data.frame(summary(stan.fit, par=c('beta', 'sigmav', 'sigmax'))$summary)

iter.rhat$id <- rep(this.id,9)

iter.rhat$year <- rep(year(hold.dat$timestamp[1]),9)

write.table(iter.rhat,file = "Summary of stan models.csv",append = TRUE,row.names = TRUE,sep=",",col.names = FALSE)

}
