# Supplementary material for: Mechanistic movement models identify continuously updated autumn migration cues in Arctic caribou
Source: Mov Ecol. 2021 Nov 1;9:54. doi: 10.1186/s40462-021-00288-0 (PMC8559358; doi:10.1186/s40462-021-00288-0)
Supplement: Supplementary file 4 — Additional file 4. Population-level model implementation. [file 40462_2021_288_MOESM4_ESM.docx]

**Additional File 4 – Population-level model implementation**

Cameron, MD, JM Eisaguirre, GA Breed, K Joly, & K Kielland. 2021. Mechanistic movement models identify continuously updated autumn migration cues in Arctic caribou. Movement Ecology. DOI: 10.1186/s40462-021-00288-0.

Example code to implement the population-level inference for the continuous time, dynamic parameter correlated random walk movement model. Corresponding R-type file available from co-authors upon request.

#############################################################################

###### Second stage MCMC of Bayesian Hierarchical dynamic CRW model ##############

########################### by: Joe Eisaguirre ###################################

####################### last updated: 3 March 2020 ###############################

#############################################################################

## In this second stage, we are gaining inference of population-level

## effects of covariates, beta[i] for the ith covariate. That is,

## beta[i,k] ~ Normal(beta[i], s2[i]) for individual k. We also gain

## inference of population-level variances, as well. That is,

## sigmav[k] ~ Normal+(sv, sv2) and sigmax[k] ~ Normal+(sx, sx2).

##############################################################################

library(rstan)

library(truncnorm)

library(ggplot2)

library(arm) # for inverse logit

setwd() # Set to folder of stanfit objects

#### ===========================

#### MCMC algorithm

#### ============================

mcmc.fun = function(bj.mat.all,

sxj.mat,

svj.mat,

n.iter,

J){

##

## Containers

##

mu.save=matrix(,nrow=length(bj.mat.all),ncol = n.iter)

bj.save=array(,dim=c(nrow(bj.mat.all[[1]]),length(bj.mat.all),n.iter))

sxj.save=matrix(,nrow(bj.mat.all[[1]]),n.iter)

svj.save=matrix(,nrow(bj.mat.all[[1]]),n.iter)

sv.save=0

sx.save=0

sv2.save=0

sx2.save=0

s2.save=matrix(,nrow=length(bj.mat.all),ncol = n.iter)

##

## priors and starting values

##

## priors

# IG(2,1) on s2, sv2, sx2

q=2

r=1

# N(0,1) on betas (and bates[k])

mu.0=0

s2.0=1

# N+(3,3^2) on sv, sx

ss.0=3

ss2.0=3^2

# IG(3,1) on sigmax[k] (same as stan model)

qx=3

rx=1

# IG(2,5) on sigmav[k] (same as stan model)

qv=2

rv=5

## starting values

mu=0

s2=1

bj=matrix(,nrow = nrow(bj.mat.all[[1]]), ncol=length(bj.mat.all))

for(i in 1:length(bj.mat.all)){

bj[,i]=apply(bj.mat.all[[i]],1,mean)

mu[i]=mean(bj.mat.all[[i]])

}

svj=apply(svj.mat,1,mean)

sv=mean(svj.mat)

sv2=1

sxj=apply(sxj.mat,1,mean)

sx=mean(sxj.mat)

sx2=1

###

### MCMC loop

###

for(k in 1:n.iter){

##

## Sample s2 (Gibbs updates)

##

for(i in 1:length(mu)){

q.tmp=J/2+q

r.tmp=1/(sum((bj[,i]-mu[i])^2)/2+1/r)

s2[i]=1/rgamma(1,q.tmp,,r.tmp)

}

##

## Sample sv2 (Gibbs updates)

##

q.tmp=J/2+q

r.tmp=1/(sum((svj-sv)^2)/2+1/r)

sv2=1/rgamma(1,q.tmp,,r.tmp)

##

## Sample sx2 (Gibbs updates)

##

q.tmp=J/2+q

r.tmp=1/(sum((sxj-sx)^2)/2+1/r)

sx2=1/rgamma(1,q.tmp,,r.tmp)

##

## Sample betas (Gibbs updates)

##

for(i in 1:length(mu)){

tmp.var=1/(J/s2[i]+1/s2.0)

tmp.mn=tmp.var*(sum(bj[,i])/s2[i]+mu.0/s2.0)

mu[i]=rnorm(1,tmp.mn,sqrt(tmp.var))

}

##

## Sample sv (Gibbs updates)

##

tmp.var=1/(J/sv2+1/ss2.0)

tmp.mn=tmp.var*(sum(svj)/sv2+ss.0/ss2.0)

sv=rtruncnorm(1,a=0,,tmp.mn,sqrt(tmp.var))

##

## Sample sx (Gibbs updates)

##

tmp.var=1/(J/sx2+1/ss2.0)

tmp.mn=tmp.var*(sum(sxj)/sx2+ss.0/ss2.0)

sx=rtruncnorm(1,a=0,,tmp.mn,sqrt(tmp.var))

##

## Sample individ-level betas (Metropolis steps)

##

for(i in 1:length(mu)){

bj.star=bj.mat.all[[i]][,k]

mh.1=dnorm(bj.star,mu[i],sqrt(s2[i]),log=TRUE)+

dnorm(bj[,i],mu.0,sqrt(s2.0),log=TRUE)

mh.2=dnorm(bj[,i],mu[i],sqrt(s2[i]),log=TRUE)+

dnorm(bj.star,mu.0,sqrt(s2.0),log=TRUE)

keep.idx=exp(mh.1-mh.2)>runif(J)

bj[,i][keep.idx]=bj.star[keep.idx]

}

##

## Sample individ-level sv's (Metropolis steps)

##

svj.star=svj.mat[,k]

for(i in 1:J){

mh.1[i]=log(dtruncnorm(svj.star[i],a=0,,sv,sqrt(sv2)))+

#log(dtruncnorm(svj[i],a=0,,ss.0,sqrt(ss2.0)))

dgamma(1/svj[i],qv,,1/rv,log=TRUE)

mh.2[i]=log(dtruncnorm(svj[i],a=0,,sv,sqrt(sv2)))+

#log(dtruncnorm(svj.star[i],a=0,,ss.0,sqrt(ss2.0)))

dgamma(1/svj.star[i],qv,,1/rv,log=TRUE)

}

keep.idx=exp(mh.1-mh.2)>runif(J)

svj[keep.idx]=svj.star[keep.idx]

##

## Sample individ-level sx's (Metropolis steps)

##

sxj.star=sxj.mat[,k]

for(i in 1:J){

mh.1[i]=log(dtruncnorm(sxj.star[i],a=0,,sx,sqrt(sx2)))+

#log(dtruncnorm(sxj[i],a=0,,ss.0,sqrt(ss2.0)))

dgamma(1/sxj[i],qx,,1/rx,log=TRUE)

mh.2[i]=log(dtruncnorm(sxj[i],a=0,,sx,sqrt(sx2)))+

#log(dtruncnorm(sxj.star[i],a=0,,ss.0,sqrt(ss2.0)))

dgamma(1/sxj.star[i],qx,,1/rx,log=TRUE)

}

keep.idx=exp(mh.1-mh.2)>runif(J)

sxj[keep.idx]=sxj.star[keep.idx]

##

## Save samples

##

mu.save[,k]=mu

s2.save[,k]=s2

bj.save[,,k]=bj

sxj.save[,k]=sxj

svj.save[,k]=svj

sv2.save[k]=sv2

sx2.save[k]=sx2

sv.save[k]=sv

sx.save[k]=sx

}

list(mu=mu.save,s2=s2.save,bj=bj.save,

sxj=sxj.save,svj=svj.save,

sx=sx.save,sv=sv.save,

sv2=sv2.save,sx2=sx2.save)

}

#### ==============================

#### end of the algorthim

#### ==============================

####

#### Load samples - This code assumes the file naming convention is year + id (ex: 2010901).

####

files = list.files(getwd(),full.names = T)

load(files[1])

samps = extract(stan.fit, pars=c('beta','sigmav','sigmax'))

samps$id = as.integer(rep(substr(list.files(getwd(),full.names = F)[1], 5, 7),

nrow(samps$beta)))

samps$yr = as.integer(rep(substr(list.files(getwd(),full.names = F)[1], 1, 4),

nrow(samps$beta)))

tmp=0

tmp.id=0

tmp.yr=0

for(i in 2:length(files)){

load(files[i])

tmp=extract(stan.fit, pars=c('beta','sigmav','sigmax'))

tmp.id = as.integer(rep(substr(list.files(getwd(),full.names = F)[i], 5, 7),

nrow(tmp$beta)))

tmp.yr = as.integer(rep(substr(list.files(getwd(),full.names = F)[i], 1, 4),

nrow(tmp$beta)))

samps$beta=rbind(samps$beta,tmp$beta)

samps$sigmav=c(samps$sigmav,tmp$sigmav)

samps$sigmax=c(samps$sigmax,tmp$sigmax)

samps$id=c(samps$id,tmp.id)

samps$yr=c(samps$yr,tmp.yr)

}

####

#### Set up the samples from individual fits

####

# number of iterations for second stage

# (same as first stage, if thinned)

n.iter=nrow(tmp$beta)

# list of individual-level beta MCMC samples

bj.mat.all=vector('list',ncol(samps$beta))

bj.mat=matrix(,nrow = length(unique(samps$id)), ncol = n.iter)

for(j in 1:length(bj.mat.all)){

for(i in 1:nrow(bj.mat)){

bj.mat[i,]=samps$beta[,j][samps$id==unique(samps$id)[i]]

}

bj.mat.all[[j]]=bj.mat

}

# matrix of individual-level sigmav MCMC samples

svj.mat=matrix(,nrow=length(unique(samps$id)), ncol = n.iter)

for(i in 1:nrow(bj.mat)){

svj.mat[i,]=samps$sigmav[samps$id==unique(samps$id)[i]]

}

# matrix of individual-level sigmax MCMC samples

sxj.mat=matrix(,nrow=length(unique(samps$id)), ncol = n.iter)

for(i in 1:nrow(bj.mat)){

sxj.mat[i,]=samps$sigmax[samps$id==unique(samps$id)[i]]

}

# number of individuals

J=dim(bj.mat)[1]

###

#### Run for data ###################

###

output.caribou = mcmc.fun(bj.mat.all,

sxj.mat,

svj.mat,

n.iter,

J)

## Summarize Betas

output.summary.df <- data.frame()

for(i in 1:7){

hold.df <- data.frame(parameter = paste("Beta",i),

type = "Beta",

est = mean(output.caribou$mu[i,]),

lower = as.numeric(quantile(output.caribou$mu[i,], c(0.05, 0.95))[1]),

upper = as.numeric(quantile(output.caribou$mu[i,], c(0.05, 0.95))[2]),

year = 2010)

output.summary.df <- rbind(output.summary.df,hold.df)

}
